# Medical Department of the University of Louisville

**Published:** 1848-02

**Authors:** 


### THE WESTERN JOURNAL.

THIRD SERIES.—VOL. I.—NO. II.

LOUISVILLE, FEBRUARY 1, 1848.

### MEDICAL DEPARTMENT OF THE UNIVERSITY OF LOUISVILLE.

The Annual Catalogue is now in the press and will soon be distributed. Meanwhile the Dean, Professor Cobb, has favored us with the following recapitulation of the present class:

| | |
|---|---|
| Kentucky | 133 |
| Tennessee | 85 |
| Mississippi | 45 |
| Alabama | 36 |
| Indiana | 30 |
| Missouri | 29 |
| Illinois | 10 |
| Texas | 9 |
| Ohio | 8 |
| Louisiana | 6 |
| Virginia | 5 |
| Arkansas | 4 |
| Georgia | 2 |
| North Carolina | 1 |
| New York | 1 |
| Iowa | 1 |
| Wisconsin Territory | 1 |
| Total | 406 |

It may not be uninteresting to many of our readers, especially the *alumni* of our University, and its predecessor, differing from it by name and charter only, to see the progressive growth of the school, and we accordingly give it:

| | | |
|---|---|---|
| A. D., 1837, | first session, | 80 |
| 1838, | second session, | 120 |
| 1839, | third session | 204 |
| 1840, | fourth session | 208 |
| 1841, | fifth session, | 262 |
| 1842, | sixth session | 189 |
| 1843, | seventh session | 243 |
| 1844, | eighth session | 286 |
| 1845, | ninth session | 345 |
| 1846, | tenth session | 349 |
| 1847, | eleventh (present) session | 406 |

In this statement we see, (with the exception of the sixth session, when there was a falling off,) a regular increase, not always at the same ratio, it is true, but a growth so steady, as to indicate the influence of substantial and permanent causes of prosperity. When this school was established, in 1837, there were, west of the mountains, but five; since that time, however, eight others have come into existence, between the Gulf of Mexico and the Lakes. One of the five has been discontinued, but the number which remain and are now in operation is thirteen. We have not, as yet, any certain account of the number of pupils, in any one of these schools except our own; but if it should only average one hundred—we have an aggregate of sixteen hundred for the Valley of the Mississippi and the Lakes, an evidence of rapidly increasing population which cannot be mistaken.

